# The quality of Finland’s postgraduate medical education: a national perspective from programme directors

**DOI:** 10.1186/s12909-026-09062-5

**Published:** 2026-03-26

**Authors:** Nea Meriranta, Sara Kaartinen, Asta Antila, Kristiina Patja, Milla Räisänen, Leila Niemi-Murola

**Affiliations:** 1 University of Helsinki, Helsinki, Finland; 2 Department of Physiatry, Helsinki University Hospital, Helsinki, Finland; 3 Clinicum, University of Helsinki, Helsinki, Finland; 4 Junior Doctors’ Association, Helsinki, Finland; 5 Clinicum, University of Helsinki and Department of General Practice and Primary Health Care, University of Helsinki and Helsinki University Hospital, Helsinki, Finland; 6 Department of Public Health, Clinicum, University of Helsinki, Helsinki, Finland; 7 Centre for University Teaching and Learning (HYPE), University of Helsinki, Helsinki, Finland; 8 Department of Anaesthesiology and Intensive Care Medicine, Helsinki University Hospital, Helsinki, Finland

**Keywords:** Postgraduate medical education, Competency-based medical education, Quality in medical education, Medical education reform, Implementation

## Abstract

**Background:**

In Finland, postgraduate medical education (PGME) is coordinated by five medical schools and delivered in training units. Following a national reform from time-based to competency-based medical education in 2020, all programme directors (PDs) collaboratively developed unified specialty-specific curricula. This study aimed to evaluate the national quality of PGME from the perspective of PDs.

**Methods:**

A survey including 36 Likert-scale and 12 open-ended questions was constructed using the World Federation for Medical Education (WFME) standards as a framework. The national electronic survey was conducted with REDCap in 2024 and e-mailed to all 253 PDs. The response rate (*n* = 82) was 35%, covering 90% of specialties.

**Results:**

Results showed broad alignment with WFME standards, particularly regarding mission clarity and competency-based structures. Variation existed in pedagogical training, mentoring resources, and assessment practices across universities. Most PDs reported having structured feedback practices and supervisory support, although structures for workplace supervision quality and trainee support varied. Only a few specialties had established clinical competency committees.

**Conclusions:**

From the PDs’ perspective, the national implementation of competency-based PGME has progressed as expected, yet notable variation remains at the grassroot level between specialties. The study reveals areas for further development, especially in harmonizing assessment practices and strengthening supervisory structures nationally, and further research is needed from the perspectives of trainees and clinical supervisors.

**Supplementary Information:**

The online version contains supplementary material available at 10.1186/s12909-026-09062-5.

## Introduction

Since the year 2020, postgraduate medical education (PGME) in Finland has undergone a significant reform, shifting from time-based towards competency-based medical education (CBME). The reform has been guided by the statute [1], which mandates national implementation in collaboration with the universities. The aim of the reform has been to provide high-quality education that meets the skill requirements of the trainees, and which in turn improves patient safety [2, 3].

Quality assurance is essential in medical education, as it ensures the graduation of competent physicians and upholds patient safety [4]. Accreditation of training hospital is the traditional method of ensuring quality of PGME, and globally several institutions and specialist associations provide this kind of services. In Finland, individual specialties have obtained international accreditation for specific teaching hospitals within their own field. In 2023, the World Federation for Medical Education (WFME) has published its updated and revised standards for PGME, designed to be flexible and applicable across diverse institutions, thereby supporting continuous quality improvement [5].

From a pedagogical quality perspective, it is essential that teaching follows the principles of constructive alignment. This means that learning objectives are clearly defined and coherently aligned with teaching content, learning activities, and assessment, so that all elements mutually reinforce one another and guide students toward meaningful deep learning [6].

Internationally, different medical education standards share similar goals: ensuring patient safety, advancing medical education quality, staying responsive to medical and societal changes, and improving healthcare delivery standards [7–9]. One prominent approach to achieving these standards has been CBME, which stands out for its focus on outcomes, emphasis on trainees' abilities, and learner-centred approach, distinguishing it from traditional time-based training [10]. However, its implementation has presented challenges, highlighting the need to evaluate the quality of PGME implementation. While PGME structures vary across Europe, different standards define what constitutes a “specialist” in the EU [11].

In Finland, there has been a traditional time-bound, master-apprentice model where traditions of formative assessment and feedback have varied at workplaces and between specialties. Due to the national reform, Finland has been one of the pioneers of CBME in Europe, along with the Netherlands and Switzerland [12, 13]. In Finland, the national reform has been implemented by using Kotter’s model, which provides a structured framework for managing curriculum change [14]. In line with this model, the sense of urgency was created by a governmental statute [1].The shared strategic vision was developed jointly by a collaborative committee of the five universities and the Ministry of Social Affairs and Health.

All fifty medical specialties have published their specific national curriculum latest in 2020 and updated versions in 2023 [15] agreeing to deliver their PGME aiming to same competencies. The number of Entrustable Professional Activities (EPA) has increased from 50 published from the year 2020 to 429 until the year 2025, reflecting the speed of the process. In Finland, universities offer education in various specialties, but the University of Helsinki (UH) is the only one that represents full range of specialties.

A self-evaluation based on WFME standards after five years provide valuable insights into the progress of the postgraduate reform, serving as a powerful tool for quality improvement. Previous research has explored PGME quality from the trainees perspective, identifying structural challenges such as excessive workload and insufficient guidance [16]. The aim of this study was to examine the national quality and implementation of PGME in Finland from the perspective of the programme directors (PDs) responsible for training, most of them professors in Finland. The PDs often collaborate with a specialist training supervisor, a senior doctor who supports and coordinates specialist training at the workplace level, working closely with the PD. However, this study focused specifically on the views and experiences of the PDs. This study used WFME standards as a framework for the survey tool, with a focus on CBME-related areas and the core components of the reform, which are outcome competencies, sequenced progression, tailored learning experiences, competency-focused instruction, and programmatic assessment [17]. We hypothesized that, five years after the implementation of the reform, substantial variation exists between universities and specialties in the implementation of the PGME reform, partly due to the autonomy of individual specialties.

## Material and methods

### Setting

In Finland, PGME is regulated by the five universities with a medical school, and the training is delivered by qualified training places in primary care and in secondary and university hospitals [18]. Before the reform, there was variation between university programs, each with a designated PD for every medical specialty. In 2020, encouraged by the statute regulation of PGME, all PDs needed to reach consensus and construct one national curriculum for each specialty [15].

### Construction of the questionnaire

This study used the WFME standards [5] as a theoretical framework to develop a self-evaluation tool. Efforts were made to make the framework measurable, allowing international comparability between different institutions. A group of four authors (L. N-M, K. P, M. R, A. A) constructed the first version in English in Autumn 2023, focusing on the essential components. The questionnaire was translated and modified into Finnish and then back translated into English by the expert group for postgraduate medical education. After the expert group reached consensus, the draft was distributed to two groups of stakeholders for further comments: first to the Board of Postgraduate Education at the UH, and then to the national Board of Postgraduate Education.

In the final setting, there were 48 questions including 36 multiple choice questions (Likert scale 1–5, 1 = strongly disagree, 2 = somewhat disagree 3 = neither agree nor disagree, 4 = somewhat agree, 5 = strongly agree) and 12 open-ended questions (Supplementary material). The questions covered topics in accordance with WFME standards: the mission of the education, curriculum, assessment, trainee welfare, supervisors, resources, quality improvement, and administrative matters [5]. We also asked specifically about patient safety.

Adherence to ethical principles was ensured through approval by the Ethics Committee of the University of Helsinki's Faculty of Medicine. Data was collected using a secure electronic questionnaire (REDCap). The respondents were asked to give their names and contact details to allow member-checking, as well as information about their pedagogical training and years of experience as PDs. We classified the pedagogical training mentioned by respondents based on the teaching skills assessment matrix used in the UH [19]. The questionnaire included a mandatory field requiring the participants to give their informed consent to store the data for 5–10 years allowing the authors to construct a follow-up survey.

### Data collection and analysis

The questionnaire was sent out to all PDs of the 50 medical specialties at each of the five Finnish universities at the turn of May and June 2024. Contact details were retrieved from a national webpage [20]. Recruitment involved direct email outreach, including a link to the survey. The authors sent two reminders, followed by a final reminder via university authorities in September 2024.

A total of 253 PDs were contacted, and 89 responses were received (35%), representing 45/50 medical specialties (90%). In some specialties, e.g. Internal Medicine, General Practice, and Anaesthesiology, there are more than one PD appointed in the university, and in some (e.g. Phoniatrics, Sports Medicine, and Forensic Medicine), one PD is responsible of PGME in two or more universities.

Of these, six PDs responded twice. After data collection, when duplicate responses were identified, the response that addressed most of the questions was included for analysis. Additionally, there was one respondent who worked as a PD in specialist dental training and was therefore not included in the study. This resulted in a final sample of 82 responses. We did not receive any answers from hand surgery, infectious diseases, neurosurgery, forensic medicine (smaller specialities), ophthalmology, and neurology (medium-sized specialities). This distribution still allowed for a balanced analysis of trends.

For the data analysis, the 50 specialities were categorized into six groups: diagnostical, conservative, operative, psychiatry, general practice, and public health medicine. We utilized the Finnish specialty classification and the categorization used in Heikkilä’s dissertation to assist in the classification process [21, 22]. In addition, we simplified the results by grouping responses as follows: “strongly disagree” and “somewhat disagree” were combined into “disagree” = 1, “neither agree nor disagree” was coded as 2, and “strongly agree” and “somewhat agree” were combined into “agree” = 3. I don’t know -answers were marked as missing.

Data was analysed using SPSS 29 to perform both descriptive and inferential statistical analyses. The median was determined for all multiple-choice question. Cross-tabulations with Fisher's exact test and the Kruskal–Wallis’s test were used to examine differences between specialty groups and universities, as well as differences based on the PD’s work experience and pedagogical education. The significance level was set at p < 0.05. Cronbach’s alpha values for different sections were as follows: questions related to the curriculum of the training program (Q11–16) 0.401, questions related to assessment (Q22–31) 0.694, and questions related to supervisors (Q38–44) 0.702.

For general text editing we utilized OpenAI´s *ChatGPT* (2025, https://chatgpt.com/). The tool was employed to enhance the fluency of the text. The qualitative content analysis of the open-ended responses was conducted using an inductive approach: meaningful units were identified from the responses and grouped into categories. ChatGPT was used as an assistive tool to support the identification of key mentions and to help organise categories. The final decisions regarding categorisation, based on primary content analysis principles and supported by AI, were made by the authors [23].

## Results

More than half of the respondents (57.3%) were women, while women constituted 47.0% of all PDs (253 in total) at the time of the survey. Nearly a third of the respondents worked in the UH, and only 9.8% worked in the University of Oulu (UO) (Table 1). The most active respondents came from the University of Eastern Finland (UEF) (45.8% of the PDs in UEF) and from UH (45.3%). We received three or more responses from the specialties of anaesthesiology and intensive care, respiratory medicine and allergology, clinical neurophysiology, paediatric neurology, paediatrics, obstetrics and gynaecology, clinical genetics, internal medicine, oncology, and general practice.

**Table 1:** Demographics of the respondents including pedagogical training and work experience as a programme director

		Specialty groups*						Universities**					
		DG	CO	OP	PSY	GP	PH	UH	UEF	UO	TAU	UTU	Total n
**Number of respondents – n**		14	36	21	5	4	2	24	22	8	12	16	82
Pedagogical education*** %													**Total %**
Excellent		0	0	0	0	0	0	0	0	0	0	0	0
Very good		7.1	11.4	4.8	0	25.0	0	8.3	4.5	25.0	16.7	0	8.6
Good		35.7	17.1	38.1	40.0	50.0	50.0	50.0	13.6	12.5	8.3	46.7	29.6
Satisfactory		28.6	25.7	28.6	20.0	0	50.0	25.0	31.8	25.0	33.3	13.3	25.9
Passable		28.6	45.7	28.6	40.0	25.0	0	16.7	50.0	37.5	41.7	40.0	35.8
**Experience in being a programme director %**													
Under 3 years		21.4	22.9	25.0	20.0	25.0	50.0	16.7	13.6	50.0	36.4	26.7	23.8
3–5 years		35.7	20.0	55.0	20.0	25.0	0	29.2	31.8	37.5	27.3	33,3	31.3
6–10 years		14.3	31.4	15.0	20.0	25.0	0	37.5	27.3	0	9.1	13.3	22.5
11–15 years		21.4	17.1	5.0	20.0	25.0	50.0	8.3	13.6	12.5	27.3	26.7	16.3
Over 15 years		7.1	8.6	0	20.0	0	0	8.3	13.6	0	0	0	6.3

^*^DG Diagnostic: Clinical physiology and nuclear medicine, clinical chemistry, clinical microbiology, clinical neurophysiology, pathology, clinical genetics and radiology. CO Conservative: Acute medicine, endocrinology, physical and rehabilitation medicine, gastroenterology, geriatrics, dermatology and allergology, cardiology, respiratory medicine and allergology, clinical haematology, child neurology, clinical pharmacology and pharmacotherapy, paediatric, phoniatrics, sports medicine, nephrology, rheumatology, internal medicine and oncology. OP Operative: Anaesthesiology and intensive care medicine, gastroenterological surgery, otorhinolaryngology, paediatric surgery, obstetrics and gynaecology, orthopaedics and traumatology, plastic surgery, oral and maxillofacial surgery. cardiothoracic surgery, urology, vascular surgery and general surgery. PSY Psychiatry: Adolescent psychiatry, child psychiatry, forensic psychiatry and psychiatry. GP General practice: general practice and occupational health. PH Public health medicine

^**^UH = University of Helsinki, UEF = University of Eastern Finland, UO = University of Oulu, TAU = Tampere University, UTU = University of Turku

^***^Excellent: Minimum 60 ECTS in teacher pedagogical studies or university pedagogy, or 55 ECTS plus special competence in medical education

Very good: Minimum 25 ECTS in teacher pedagogical studies or university pedagogy, or 20 ECTS plus special competence in medical education

Good: Minimum 10 ECTS in teacher pedagogical studies or university pedagogy, or 5 ECTS plus special competence in medical education

Satisfactory: Minimum 5 ECTS in teacher pedagogical studies or university pedagogy, or special competence in medical education

Passable: Limited pedagogical studies or training, e.g., individual training sessions or events

Major differences occurred in the amount of pedagogical education between PDs working in the five universities. Over half of the PDs in UH and in the university of Turku (UTU), had ten credits or more pedagogical education compared with 18.5% in UEF. In contrast, UEF had the most of very experienced (over 15 years) PDs and UO had the least experienced PDs of whom 87.5% had experience five years or less (Table 1).

### Curriculum and assessment

On a national level, basic structures of competency-based PGME seemed to be well implemented. Most of the respondents agreed (93.9%) that their specialties had a clear mission of the training programme and 75.6% that the competency outcomes were based on international guidelines (Table 2). The PDs, having more than 15 years of experience, were more critical of the latter item compared to those having less experience.

**Table 2:** Selected questionnaire items, based on WFME standards, showing general agreement (median = 3) among Finnish programme directors in 2024

	**Question**	Agreement-%*	Statistical analysis**
10	The training program for postgraduate medical education has a clear mission	93.9	
**11**	**Competency outcomes are based on international guidelines/framework, e.g. European Education Requirements (ETR)**	**75.6**	***F*: Experience in being a programme director *p* = 0.017** ***F*: Specialties *p* < 0.001** ***KW*: Specialties *p* = 0.007**
12	Knowledge, skills, and attitudes required by trainees in our field are included in competency outcomes	95.1	
13	There are descriptions of the requirements for entry-level knowledge, skills, and attitudes for those starting postgraduate education	56.1	
14	During postgraduate education in our field, opportunities for research work either as part of the training program or during absence for the completion of a scientific postgraduate degree are available	93.9	
15	There are regular follow-up discussions mapping the progress of the trainees towards the intended educational outcomes	93.9	
16	The feedback discussions are structured and assess the achievement of competence goals	95.1	
22	Our specialty has constructed programmatic assessment for postgraduate education	80.5	
23	Programmatic assessment is based on the intended learning outcomes in our field	85.4	
24	During postgraduate education in our field, mainly formative assessment during learning process is used to support learning	70.7	
26	Trainees progress is reviewed at regular intervals	67.1	
27	It is possible to identify trainees in need of additional support in our specialty	86.6	
30	There are ways to support those trainees who have an identified need for enhanced support in our field	72.0	
**34**	**We can provide mentoring for trainees in our field**	**73.2**	***F*: Universities *p* = 0.018** ***F*: Specialties *P* = 0.018** ***KW*: Specialties *p* = 0.026**
35	We can provide professional guidance for trainees in our field	54.9	
38	Pedagogical training is available for teachers and clinical supervisors in our specialty	87.8	
39	Earmarked time is available for implementing supervision	57.3	
40	Earmarked time is available for implementing observations and assessment	54.2	
**41**	**We have a system with which we ensure the quality of supervision in the workplace**	**79.3**	***F*: Universities *p* = 0.029** ***KW*: Universities *p* = 0.022**
43	We have a defined code of conduct for those participating in the clinical supervision	78.0	
44	Supervisors’ continuous professional pedagogical development is supported and valued	72.0	
**45**	**Our specialty has the prerequisites for theoretical learning in the workplace (facilities, computers, internet)**	**92.7**	***F*: Universities *p* = 0.006** ***KW*: Universities *p* = 0.012**
46	Our specialty has the prerequisites for learning practical skills in the workplace (e.g. simulations, skill stations and equipment)	74.4	
47	We have basic prerequisites for learning in the workplace and patient care (facilities, equipment, staff)	82.9	
48	We collect feedback to develop training and improve its quality	72.0	
**50**	**We respond to the feedback by describing actions based on the feedback received**	**61.0**	***F*: Universities *p* = 0.010** ***KW*: Universities *p* = 0.012** ***KW*: Specialty groups *p* = 0.021**
**51**	**The trainees need for guidance and mandate for independent practice is based on entrustment decisions**	**41.5**	***F*: Specialties *p* = 0.028**
52	We have identified risks to patient safety related to the trainees on-going training and activities	75.6	
55	Sufficient administrative structures exist in our specialty to support the organization of training (staff, equipment, software)	59.8	

*F*  Fisher’s exact test, *KW*  Kruskal–Wallis test

^*^Agreement-% represents the percentage of respondents who selected either “strongly agree” or “somewhat agree” on the original 5-point Likert scale. For statistical analysis, these responses were combined and recoded as “agree” = 3

^**^Statistically significant differences between groups displayed and highlighted in bold

According to qualitative content analysis the competency outcomes were unequally distributed to trainees, clinical supervisors, and stakeholders (Table 4). Distribution of information seemed more systematic and structured to trainees than to supervisors.

Trainees accessed information about the intended competency outcomes mainly via the national websites (51 mentions) and personal discussions with PDs and specialist training supervisors (33 mentions). The most frequent information channel for supervisors was also the national websites (38 mentions) and personal discussions (25 mentions). Stakeholders (hospital administration) were primarily informed in person by the PDs/specialist training supervisor (13 mentions) and through meetings (10 mentions).

When asked about the primary type of assessment used in their specialty (formative or summative), respondents gave conflicting answers, indicating the use of both. Respondents with less than very good pedagogical training (i.e., fewer than 25 ECTS in teacher pedagogical studies or university pedagogy, or 20 ECTS plus a special competence in medical education) were more likely to answer affirmatively with both options (Tables 2 and 3).

**Table 3:** Selected questionnaire items, based on WFME standards, showing general disagreement (median = 1) or mixed opinions (median = 2) among Finnish programme directors in 2024

	**Question**	**Median**	Disagreement-%*	Statistical analysis**
**25**	**During postgraduate education in our field, mainly summative assessment is used e.g. patient exam)**	**2**	**36.6**	***F*: Specialty groups *p* = 0.011**
28	There is a designated working group in our field that processes the data of individual trainee’s progress and gives possible recommendations about the need for additional support	1	54.9	
29	There is a designated working group in our field that prepares a program to improve the trainee's performance	1	56.1	
**31**	**There is a designated working group in our field that has the authority to suspend a trainee’s training (and possibly provide career guidance)**	**1**	**61.0**	***KW*: Specialty groups *p* = 0.044**
42	We have a quality system allowing trainees to give feedback on the performance of their supervisors	2	41.5	
49	We publish the results of the collected feedback regularly	2	40.2	
**54**	**We have a separate budget for the development of postgraduate education**	**1**	**54.9**	***F*: Universities *p* < 0.001** ***KW*: Universities *p* < 0.001**

*F * Fisher’s exact test, *KW * Kruskal–Wallis test

^*^Disagreement-% represents the percentage of respondents who selected “strongly disagree” or “somewhat disagree” on the original 5-point Likert scale. For statistical analysis, these responses were combined and recoded as “disagree” = 1

^**^Statistically significant differences between groups displayed and highlighted in bold

General agreement existed on item stating that the feedback discussions are structured and do assess the achievement of competence goals (Table 2). About half of the respondents (52.4%) reported trainees having feedback discussions more than once a year, with the highest frequency among those in operative specialties (70.6%). Nearly a third (31.7%) of all respondents had feedback discussions once a year.

In open-ended responses (Table 4), PDs highlighted that learning was primarily supported by close supervision and personal guidance (45 mentions).

**Table 4:** Results of the qualitative content analysis

**Question**	N	Codes (number of mentions)	Quotes from the data
Q17 In which way have the competency outcomes been communicated to trainees?	76	National websites (including EPAs, curriculum guide, assessment guide): 51 Communication between programme directors, specialist training supervisor, and trainees: 33 ELSA (a digital follow-up and assessment platform) and logbooks: 23 Seminars: 10	*“In the curriculum guide, the assessment guide, and the EPA descriptions”* *“Logbook, EPAs, initial and final discussions with the supervisor, training discussions with the programme director and specialist training supervisor.”* *“Learning objectives are reviewed during the start seminar. They are part of the assessment process and also linked to ELSA”*
Q18 In which way have the competency outcomes been communicated to supervisors?	74	National websites (including EPAs, curriculum guide, assessment guide): 38 Communication between programme directors, specialist training supervisor, and trainees: 25 ELSA (a digital follow-up and assessment platform) and logbooks): 18 Seminars: 23	*“Curriculum guide and EPAs, training-related meetings”* *“In feedback discussions, the logbook is used to review progress in competence development. The specialty is so small that the programme director and specialist training supervisors provide daily supervision….”* *“Supervisors have relatively poor insight into this. Assessments often come as a surprise – they are generally unaware of the learning objectives unless the trainee specifically communicates them… This area requires significant development.”*
Q32 In what way have you communicated the programmatic assessment to stakeholders?	49	In person from the responsible trainer/specialist training supervisor (e.g. during training place evaluations and agreement processes): 13 Meetings (clinical staff/trainer meetings): 10 Trainings: 6	*”Probably not in any way.”* *”Through site visits, educational collaboration seminars, specialist training supervisor, and regional chief doctors in general practice training.”* *”There is no formal written assessment programme. However, regular discussions are held with local training supervisors to ensure the suitability of the training sites.”*
Q20 How is trainees learning supported in training places/training hospitals?	70	Close supervision and personal guidance: 45 feedback discussions and assessments: 20 Theoretical training in workplace and participation in congresses/courses: 19 documentation such as logbooks and EPAs: 11 planning of clinical rotations/placements: 10	*”Through guidance discussions following the master–apprentice tradition.”* *“Biannual follow-up discussions to review progress and attainment of EPA objectives”* *Weekly on-site supervision is key. Regular in-house training sessions provided by specialist training supervisor.”* *“Structured rotation plan, regular senior support”*
Q33 In what way will you back up the trainees needing enhanced support?	59	Personal guidance (discussions, additional support etc.): 40 Extension of specialization period/scheduling adjustments/job description modifications: 21 Other support (mentoring, psychologist, supervision of work): 7 Literature, workshops, additional training: 11	*”Personal additional support.”* *”Discussion, extension of the specialization period.”* *“…searching for appropriate training or literature together…”*

### Resources and supervisors

On university level, essential components of constructing PGME were mostly in place. Vast majority of respondents (87.8%) felt that universities endorse PGME by organizing pedagogical training for clinical supervisors and clinical supervisors’ continuous professional pedagogical development is supported and valued (72.0%). General agreement existed on having adequate resources for both theoretical and clinical training in the workplace (Table 2).

PDs reported that time has been allocated to implementing supervision observations and assessments at the workplace. A system to ensure the quality of supervision at the workplace was acknowledged by most universities, but UEF and UO were not unanimous in stating that they have such a system. Most universities had a quality system for trainees to give feedback on the performance of their supervisors, and universities also responded to the trainees (Table 2).

### Trainee welfare

Universities differed in how mentoring was resourced. According to the PDs, 93.8% from UTU reported that mentoring has been provided. In contrast, in HU 27.3% stated that mentoring has not been provided.

Most of the respondents from different specialties had ways to recognize (86.6%) and support (72.0%) those trainees who have an identified need for enhanced support (Table 2). The most frequently mentioned methods to support the trainees were individual support and guidance (40 mentions) and extension of specialization period and regulation of workload and job description (21 mentions).

The appointment of designated working groups (e.g. Clinical competency committees) responsible for handling specialists-in-training information, identifying their needs, and recognizing the need for interruption or career counselling varied across specialties. Only ten specialties were unanimous that they had introduced clinical competency committees (Table 3) and less than half of the PDs agreed that entrustment decisions were used to determine the need for guidance and mandate for independent practice (Table 2).

## Discussion

The implementation of national PGME started from the scratch in hospital specialties in Finland in 2020. This study found that from the perspective of the PDs the implementation of the structures of competency-based PGME has nationally proceeded as expected between 2020 and 2024. All fifty medical specialties have created a national curriculum, and most have introduced at least three EPAs. Most specialties indicated having means to recognize and support trainees with an identified need for enhanced support even though clinical competency committees were still lacking. Majority of the PDs reported that the universities provide the required resources such as pedagogical training for supervisors, time for evaluations at the workplaces, and a quality control system including a feedback questionnaire for trainees. Although national implementation has proceeded well, notable variation exists at the grassroots level between specialties.

Before the reform, Finland had a traditional, time-based master-apprentice education and all five universities had their own, somewhat differing programs delivered in training places. After five years of the reform, this implementation evaluation based on WFME standards provided a way to check whether the nationally similar PGME programs are being carried out as planned [24, 25] and what are the major concerns to be addressed. Autonomy of the 50 medical specialties has been the major self-evaluation challenge of this reform, and the questionnaire also aimed to assist the PDs to benchmark their achievements with global (WFME) standards [5].

### Curriculum and assessment

Most specialties, gratifyingly, have reached a shared understanding of the outcomes of PGME which are based on international recommendations. This step was facilitated by the fact, that most specialties had constructed their existing local curriculum based on European Training Requirements [26] and the CanMeds framework is widely acknowledged in Finnish PGME [27].

In contrast to common curriculum creation, the introduction of meaningful workplace-based assessment appeared to be more difficult to achieve [28]. The open-ended answers provided the observation that trainees got more structured information on learning outcomes compared to supervisors who are responsible for the assessment at the workplace. This may partly be related to varying pedagogical education provided by the universities. To facilitate the introduction of workplace-based assessment the information must be more equally delivered, and the trainees need to take more responsibility for their own training [13, 28].

From the perspective of constructive alignment [6], our findings suggested that assessment practices are not yet fully aligned with learning objectives and teaching methods. While most respondents agreed that formative assessment [29] is widely used, many also agreed that mainly summative assessment is used which makes the situation somewhat unclear. One possible explanation for this contradiction is the lack of pedagogical training and confusion on terminology among PDs; another may be that along with EPAs final exams are still required in all specialties, reinforcing a more traditional assessment structure [30]. In competency-based PGME formative assessment during the learning process is crucial in aim to provide feedback for trainees that supports them in becoming aware of their own competencies and the gaps within them, as well as enable them to develop their skills [31]. This is an important skill in their future work as medical experts. Although the emphasis in CBME is in formative assessment, summative assessment can complement it effectively.

What comes to the pedagogical competence of programme directors, it is plausible that this influences how competency-based principles are understood and translated into everyday training practices. Programme directors play a central role in implementing educational change in postgraduate medical education [32]. In a context of substantial specialty autonomy and varying university-level pedagogical requirements, differences in pedagogical training may partly contribute to uneven implementation of the reform.

Trainee perspectives from the biannual Training Place Survey, conducted by the Finnish Junior Doctors’ Association and the Finnish Medical Association, have offered further insight into the implementation of assessment practices. In 2023, 49% of trainees agreed fully or somewhat with the statement: “I collect feedback together with my supervisor that supports my performance and professional competence” [33]. This indicated that about half of the trainees experience structured feedback practices. However, almost all the PDs reported that feedback discussions are structured and assess the achievement of competence goals. This discrepancy suggests a potential gap between trainee experience and PD perception; while PDs may consider the feedback process well established, trainees may not consistently perceive it as such.

From the trainee perspective, 52% reported receiving supervision at least monthly [33]. These figures align with PDs’ responses indicating that earmarked time is available for implementing supervision. With regard to the follow-up and feedback discussions, some respondents found the terminology unclear, reflecting the ongoing nature of the implementation process. Mostly PDs reported having annual or biannual follow-up discussions with the trainees, yet some reported that feedback discussions are taking place after every clinical rotation (from one to six months) or in every assessment at the workplace.

Moreover, most specialties reported having introduced programmatic assessment [34–36] but just a few of them had introduced clinical competency committees. Before the reform, all decisions were made by the PD, and there has been reluctancy to complicate the process of graduation [37]. Based on the survey it seemed that PDs mostly make the final decisions without competency committees. A similar phenomenon has been reported in Canada, where the implementation of CBME has increased the assessment burden [38].

### Resources and supervisors

According to the results, the universities have provided the essential elements of PGME. Most PDs agreed that the prerequisites for theoretical teaching and learning at the workplace have been provided. Quality of education in primary care and secondary hospitals has been secured with formal specialist training placement agreements. Many of these structures existed before the reform.

Before the reform, pedagogical education was a bottleneck, as it was mostly available for undergraduate teachers and university staff/personnel in hospital specialties. This was reflected in responses, where PDs with very good qualifications described their practices more precisely. In the past five years, basic pedagogical training has been extended more generally to clinical supervisors as well [37]. Since teaching and learning in the workplace differ from undergraduate education, three-credit courses have been developed to meet the needs of clinical educators [39, 40]. These courses have been designed based on the requirements for special competence in medical education [41]. The outlook was positive, as continuous pedagogical development for supervisors is widely supported across specialties.

### Trainee welfare

Most specialties reported having means to recognize and support trainees in need of additional support even though no designated group existed for this. Ideally, the practice would include reviewing individual trainee’s progress data with the aim of providing recommendations about the need for additional support. Thus, it may be assumed, that these practices to support trainees existed before the reform, and they are not based on structured observations and learning analytics [28]. Many of the reported ways of additional support were workplace-related such as individual support and guidance, strengthening senior support, and regulation of workload.

The five Finnish universities differed in the delivery of mentoring for trainees. One university (UTU) has had a special program as is evident in that almost all PD’s from UTU agreed with question on providing mentoring. The other universities seemed not to have a unified system, and differences existed between specialties. In Finland, there has been different stakeholders related to and providing mentoring such as Duodecim and different specialty associations which may partly explain the variety. Mentoring, understood as an equal partnership between the mentor and the mentee, plays an important role in professional development [42]. In contrast, supervision is more structured and focuses on providing guidance and feedback on personal, professional, and educational development within the context of ensuring safe patient care [43].

According to the Training Place Survey, only 14% of trainees reported having a designated mentor and 25% a designated tutor, who could also act as a mentor [33]. The level of mentoring appeared lower than expected based on PD responses, suggesting that mentoring might be less consistently available from the trainee perspective. However, the phrasing “designated mentor” may have narrowed the interpretation, excluding informal or non-assigned mentoring relationships that PDs might still have considered part of the mentoring structure.

### Strengths and limitations

The Finnish PGME reform has been internationally unique in the sense that the urge for the reform was created by the statute and the national strategic vision has been constructed and delivered in collaboration of the five universities and the Ministry of Health and social services [1, 15]. In the implementation of CBME based PGME Finland has stood in the forefront and thus provides a valuable example along with the early adopters including Netherlands and Canada [12, 44].

Our response rate was 35%, which we considered a good result for this type of survey. However, the study captured only the perspectives of PDs. Other stakeholder groups may have different experiences and views on how the reform functions in practice, and these were beyond the scope of the present study. In addition, not all specialties were represented, and participation varied significantly between universities. Notably, approximately 40% of PGME in Finland takes place at the UH, which had a response rate of 45%, suggesting that the results likely reflect the general reality in Finland. However, a positive response bias may have occurred, as those who ultimately responded to the survey were likely the ones most familiar with the reform, best understood its concepts, and were most motivated to implement it.

A key strength of the study was that, in addition to responding to statements, participants had the opportunity to elaborate on their experiences through open-ended answers. This combination of qualitative and quantitative data provided a more comprehensive understanding of their experiences and perspectives, further enhancing the study’s validity.

Due to lack of or only a few responses from some specialties we grouped the specialties with the aim of finding differences between the groups. However, also these educationally meaningful groups were somewhat differing in size which may have resulted in lack of power in the comparison analysis. Moreover, the phase of implementation of competency-based PGME may have varied more between the specialties and/or working places than specialty groups. In addition, some more subtle differences might have been undetected due to categorization of the Likert answers to “agree” and “disagree”, however, this was also necessary for comprehensive statistical analyses.

The differences between universities regarding pedagogical education and work experience of the PDs might have affected the results in the sense of understanding the questionnaire items. In the UH, the minimum requirement for PDs is ten credits of formal pedagogical education which clearly was not reached in the University of Tampere (TAU) and UEF. Although the authors aimed to create an unambiguous questionnaire, some participants may have struggled to fully comprehend certain items, as also indicated in the open-ended responses. The recent reform has introduced new concepts, whose translation into Finnish was not entirely easy. Additionally, the lack of established terminology has further complicated understanding. The curriculum-related items (Q11–16) showed low internal consistency, suggesting they reflect multiple aspects of curricular implementation rather than a single construct. Cronbach’s alpha improved towards the end of the questionnaire, which may be due to the concepts being more familiar in the later sections. As the analyses were conducted at the item level rather than using composite scores, this limitation does not undermine the interpretation of the individual items. In the future, the questionnaire should be further developed to improve the measurement of the quality of competency-based PGME.

## Conclusions

From the PDs’ perspective, the national implementation of competency-based PGME in Finland has progressed as expected over the past five years. Universities have succeeded in establishing competency-based practices across specialties, outcome competencies are defined, and formative assessment is gradually being introduced. Programmatic assessment remains a challenge.

While some variation between specialties was observed, as we anticipated, only minor differences between specialty groups indicated that CBME is feasible in all specialty groups. WFME standards provide a valuable framework for guiding a reform towards CBME and supporting quality improvement in PGME.

This study provides a foundation for future follow-up research in Finland, as well as a comparison for other countries involved in a similar reform. In addition, the PDs’ perspective presented here should be complemented with input from trainees, clinical supervisors, and other stakeholders to gain a more comprehensive understanding of the reform implementation. The findings also point out the importance of harmonising national expectations for pedagogical competence among programme directors, as variation in pedagogical training may influence the success and consistency of implementing national competency-based PGME. In future, the national progress would also benefit for sharing best practices and innovation diffusion between PDs.

## Supplementary Information

Supplementary Material 1

## Data Availability

The dataset generated and analysed during the current study is not publicly available due to privacy and confidentiality restrictions, as the survey data contain potentially identifiable information about participants.

## Abbreviations

PGME: Postgraduate medical education
PD: Programme director
WFME: The World Federation for Medical Education
CBME: Competency-based medical education
EPA: Entrustable professional activity
UH: The University of Helsinki
UO: The University of Oulu
UEF: The University of Eastern Finland
UTU: The University of Turku
TAU: Tampere University
