# Supplementary material for: The quality of Finland’s postgraduate medical education: a national perspective from programme directors
Source: BMC Med Educ. 2026 Mar 26;26:720. doi: 10.1186/s12909-026-09062-5 (PMC13141363; doi:10.1186/s12909-026-09062-5)
Supplement: Supplementary file 1 — Supplementary Material 1 [file 12909_2026_9062_MOESM1_ESM.docx]

Supplementary material – Questionnaire for programme directors using the World Federation for Medical Education (WFME) standards as a framework

Q1 I am aware that the answers I provide can be linked to me as a respondent

Q2 I give my consent for my answers to be used for research purposes.

Q3 I give my consent for my answers to be stored for the purpose of follow-up research.

Q4 Name of the programme chair who filled this questionnaire: ____________________________

Q5 Specialty: ____________________________________Q6 University_________________________

Q7 e-mail: _________________________________

Q8 Pedagogical education_____________________________________________________________

Q9 Number of years worked as the programme chair: ___________________________________

**Mission**

Q10 The training program for postgraduate medical education has a clear mission

Strongly disagree, Disagree, Neither agree nor disagree, Agree, Strongly agree, Don't know/Cannot say

**Curriculum**

Q11 Competency outcomes are based on international guidelines/framework, e.g. European Education Requirements (ETR)

Strongly disagree, Disagree, Neither agree nor disagree, Agree, Strongly agree, Don't know/Cannot say

Q12 Knowledge, skills, and attitudes required by residents in our field are included in competency outcomes.

Strongly disagree, Disagree, Neither agree nor disagree, Agree, Strongly agree, Don't know/Cannot say

Q13 There are descriptions of the requirements for entry-level knowledge, skills, and attitudes for those starting postgraduate education

Strongly disagree, Disagree, Neither agree nor disagree, Agree, Strongly agree, Don't know/Cannot say

Q14 During the postgraduate education in our field, opportunities for research work either as part of the training program, or during absence for the completion of a scientific postgraduate degree are available

Strongly disagree, Disagree, Neither agree nor disagree, Agree, Strongly agree, Don't know/Cannot say

Q15 There are regular follow-up discussions mapping the progress of the residents towards the intended educational outcomes

Strongly disagree, Disagree, Neither agree nor disagree, Agree, Strongly agree, Don't know/Cannot say

Q16 The feedback discussions are structured and assess the achievement of competence goals

Strongly disagree, Disagree, Neither agree nor disagree, Agree, Strongly agree, Don't know/Cannot say

Q17 In which way have the competency outcomes been communicated to residents?

__________________________________________________________________________

Q18 In which way have the competency outcomes been communicated to supervisors?

__________________________________________________________________________

Q19 In addition to learning on the job, what kind of learning methods are used in the field specialist training?

__________________________________________________________________________

Q20 How is the residents’ learning supported in training places/training hospitals?

__________________________________________________________________________

Q21 How often do you have these follow-up discussions?

__________________________________________________________________________

**Assessment**

Q22 Our specialty has a constructed programmatic assessment for postgraduate education

Strongly disagree, Disagree, Neither agree nor disagree, Agree, Strongly agree, Don't know/Cannot say

Q23 Programmatic assessment is based on the intended learning outcomes in our field

Strongly disagree, Disagree, Neither agree nor disagree, Agree, Strongly agree, Don't know/Cannot say

Q24 During postgraduate education in our field, mainly formative assessment during learning process is used to support learning

Strongly disagree, Disagree, Neither agree nor disagree, Agree, Strongly agree, Don't know/Cannot say

Q25 During postgraduate education in our field, mainly summative assessment is used (e.g. patient exam)

Strongly disagree, Disagree, Neither agree nor disagree, Agree, Strongly agree, Don't know/Cannot say

Q26 Residents’ progress is reviewed at regular intervals.

Strongly disagree, Disagree, Neither agree nor disagree, Agree, Strongly agree, Don't know/Cannot say

Q27 It is possible to identify residents in need of additional support in our specialty.

Strongly disagree, Disagree, Neither agree nor disagree, Agree, Strongly agree, Don't know/Cannot say

Q28 There is a designated working group in our field that processes the data of individual resident’s progress and gives possible recommendations about the need for additional support

Strongly disagree, Disagree, Neither agree nor disagree, Agree, Strongly agree, Don't know/Cannot say

Q29 There is a designated working group in our field that prepares a program to improve the resident's performance

Strongly disagree, Disagree, Neither agree nor disagree, Agree, Strongly agree, Don't know/Cannot say

Q30 There are ways to support those residents who have an identified need for enhanced support in our field

Strongly disagree, Disagree, Neither agree nor disagree, Agree, Strongly agree, Don't know/Cannot say

Q31 There is a designated working group in our field that has the authority to suspend a resident’s training (and possibly provide career guidance)

Strongly disagree, Disagree, Neither agree nor disagree, Agree, Strongly agree, Don't know/Cannot say

Q32 In what way have you communicated the programmatic assessment to stakeholders (e.g. service system)

__________________________________________________________________________

Q33 In what way will you support the residents needing enhanced support?

__________________________________________________________________________

**Resident welfare**

Q34 We can provide mentoring for residents in our field

Strongly disagree, Disagree, Neither agree nor disagree, Agree, Strongly agree, Don't know/Cannot say

Q35 We can provide professional guidance for residents in our field.

Strongly disagree, Disagree, Neither agree nor disagree, Agree, Strongly agree, Don't know/Cannot say

Q36 In what way will you follow up residents’ welfare at work?

__________________________________________________________________________

Q37 What kind of influencing opportunities do the residents in your field have in their specific workplaces?

__________________________________________________________________________

**Supervisors**

Q38 Pedagogical training is available for teachers and clinical supervisors in our specialty

Strongly disagree, Disagree, Neither agree nor disagree, Agree, Strongly agree, Don't know/Cannot say

Q39 Earmarked time is available for implementing supervision

Strongly disagree, Disagree, Neither agree nor disagree, Agree, Strongly agree, Don't know/Cannot say

Q40 Earmarked time is available for implementing observations and assessment

Strongly disagree, Disagree, Neither agree nor disagree, Agree, Strongly agree, Don't know/Cannot say

Q41 We have a system with which we ensure the quality of supervision in the workplace

Strongly disagree, Disagree, Neither agree nor disagree, Agree, Strongly agree, Don't know/Cannot say

Q42 We have a quality system allowing residents to give feedback on the performance of their supervisors

Strongly disagree, Disagree, Neither agree nor disagree, Agree, Strongly agree, Don't know/Cannot say

Q43 We have defined code of conduct for those participating in the clinical supervision

Strongly disagree, Disagree, Neither agree nor disagree, Agree, Strongly agree, Don't know/Cannot say

Q44 Supervisors’ continuous professional pedagogical development is supported and valued

Strongly disagree, Disagree, Neither agree nor disagree, Agree, Strongly agree, Don't know/Cannot say

**Resources**

Q45 Our specialty has the prerequisites for theoretical learning in the workplaces (facilities, computers, internet)

Strongly disagree, Disagree, Neither agree nor disagree, Agree, Strongly agree, Don't know/Cannot say

Q46 Our specialty has the prerequisites for learning practical skills in the workplaces (e.g. simulations, skill stations and equipment)

Strongly disagree, Disagree, Neither agree nor disagree, Agree, Strongly agree, Don't know/Cannot say

Q47 We have basic the prerequisites for learning at workplace and patient care (facilities, equipment, staff)

Strongly disagree, Disagree, Neither agree nor disagree, Agree, Strongly agree, Don't know/Cannot say

**Quality improvement**

Q48 We collect feedback to develop training and improve its quality

Strongly disagree, Disagree, Neither agree nor disagree, Agree, Strongly agree, Don't know/Cannot say

Q49 We publish the results of the collected feedback regularly

Strongly disagree, Disagree, Neither agree nor disagree, Agree, Strongly agree, Don't know/Cannot say

Q50 We respond to the feedback by describing actions based on the feedback received

Strongly disagree, Disagree, Neither agree nor disagree, Agree, Strongly agree, Don't know/Cannot say

**Patient safety**

Q51 We have identified risks to patient safety related to the residents’ on-going training and activities

Strongly disagree, Disagree, Neither agree nor disagree, Agree, Strongly agree, Don't know/Cannot say

Q52 The residents’ need for guidance and mandate for independent practice is based on entrustment decisions

Strongly disagree, Disagree, Neither agree nor disagree, Agree, Strongly agree, Don't know/Cannot say

Q53 How do you ensure patient safety in regards with the residents’ clinical work?

__________________________________________________________________________

**Administration**

Q54 We have a separate budget for the development of postgraduate education

Strongly disagree, Disagree, Neither agree nor disagree, Agree, Strongly agree, Don't know/Cannot say

Q55 Sufficient administrative structures exist in our specialty to support the organization of training (staff, equipment, software)

Strongly disagree, Disagree, Neither agree nor disagree, Agree, Strongly agree, Don't know/Cannot say

Q56 The following stakeholders are represented in the administration of postgraduate education in our field

- Representative of the service system (chief physician or equivalent)
- Specialist training supervisor /Medical Officer in Charge of Training
- Representative of local trainers
- Other representative of the health service system (e.g., nursing staff)
- Representative of university academic administration
- None of the above

Q57 How has your field ensured the transparency of the administration of postgraduate education?

__________________________________________________________________________

Q58 Additional comments

__________________________________________________________________________
